# An Investigation Into Contaminated Waste Composition in a University Dental Clinic: Opportunities for Sustainability in Dentistry

**DOI:** 10.1002/cre2.70015

**Published:** 2024-10-29

**Authors:** Samuel Yeoh, Yani Bourdamis, Adam Saker, Noah Marano, Liam Maundrell, Poornima Ramamurthy, Dileep Sharma

**Affiliations:** ^1^ College of Medicine and Dentistry James Cook University Cairns Queensland Australia; ^2^ School of Health sciences The University of Newcastle Ourimbah New South Wales Australia

**Keywords:** clinical waste, environmental sustainability, university dental clinic

## Abstract

**Objectives:**

Many international dental organizations have been advocating for sustainable practices in dentistry, whereby significant reductions in environmental impacts are needed. The aim of this study was to analyze dental clinical waste in a university clinic setting to explore opportunities for sustainable practices.

**Material and Methods:**

Fifty dental units (chairs) that are routinely used in delivery of dental treatment and involved supervising clinicians, dental students, and patients were randomly selected, and the clinical waste generated was collected, segregated, and weighed. Statistical analysis was performed to analyze differences in waste production based on treatment performed.

**Results:**

The mean waste production generated by each chair was 81.4 g of aprons, 56.2 g of gloves, 17.2 g of masks, 24.0 g of sterile wrappings, 48.8 g of other plastics, 100.8 g of cellulose‐based items, and 25.8 g of miscellaneous items. Higher waste was generated from the chairs performing endodontic procedures when compared with examinations. A potential annual greenhouse gas saving of approximately 10 kg CO_2_e per year (when one patient is treated daily) can be achieved if sterile wrapping plastics were to be recycled.

**Conclusions:**

Simple yet achievable opportunities for efficient clinical waste management at university clinics exist, which in turn will increase environmental sustainability in the post‐COVID‐19 era. Increased awareness and incentives for sustainable measures could potentially enhance the possibility of wider adoption of ecofriendly approaches.

**Abbreviations**
GHG: greenhouse gas
JCU: James Cook University
PPE: Personal protective equipment

## 1 Introduction

Healthcare facilities play a critical role in treating patients and handling outbreak of diseases to maintain societal health and welfare. This industry represents a large portion of the economy with significant workforce, facilities, resources, and energy expenditure. Healthcare industry, particularly in developed countries, have a large climate impact, with a significant contribution of 3%–8% to the overall national greenhouse gas (GHG) emissions (Brown, Buettner, and Canyon 2012). With the increasing population, consumption of resources, and greenhouse gas emissions, environmental sustainability is an essential strategy that must be implemented. Environmental sustainability is seen as services that are specifically planned, financed, and delivered to best meet the needs of both the current population and future generations (Brown, Buettner, and Canyon 2012). Sustainability in dental practice can help integrate the oral health profession to environmental and social responsibility. Not only will eco‐friendly dentistry benefit the environment but also become financially viable along with the reduction in waste production, energy consumption, and pollution (Grose et al. 2018).

Biomedical waste is defined as any “waste that is generated during the diagnosis, treatment or immunization of human beings or animals” (Arora, Mittal, and Dogra 2017). The World Health Organization outlines goals for healthcare systems wherein minimizing and adequately managing waste, including hazardous chemicals and reducing emissions of greenhouse gases are at its forefront (WHO 2017). A recent report by Academy of Royal Medical Colleges recognized the need for clinicians to be “innovative to tackle the huge financial challenges” (Maughan and Gibbs 2014). The report identified a range of behaviors to achieve this, among which the need for “a change in culture where doctors resolve to eradicate waste” was highlighted (Maughan and Gibbs 2014). International dental organizations like the World Dental Federation (FDI) supported by the Australian Medical and Dental Associations (AMA and ADA) have also been advocating several approaches toward sustainability in dentistry (AMA 2019; Duane et al. 2019). Furthermore, the AMA has called on health professionals to lead the way toward facilitating a sustainable healthcare sector whereby significant reductions in environmental impacts can be achieved (AMA 2019).

Climate change, despite being a global issue, cannot be resolved through policy changes alone but contributions toward carbon footprint, at individual level, needs to make better and more informed decisions, to meet the goal of environmental sustainability (Grose et al. 2018). In addition to commitments made by professional bodies, dental practices, as a healthcare provider, is yet to make significant strides toward sustainability. Broad goals set with a top‐down approach has been less effective in fostering meaningful changes. Rather, a more community‐based approach which targets local contexts is needed as they are tailored toward specific dental settings. Individual and small‐scale initiatives to make measurable yet scalable changes could be the focus, as the first stage in creating widespread eco‐friendly dental clinics. In this context, engaging the dental community, in particular, the private dental clinics, larger public dental hospitals, and university clinics are critical (Duane et al. 2019).

The move towards environmentally sustainable dental practices can be achieved using the 4Rs approach: rethink, reduce, reuse, and recycle (Arora, Mittal, and Dogra 2017). The first step in this move towards eco‐friendly practice is to re‐evaluate current procedures and protocols. Reducing the amount of waste production and reusing items needs to be implemented into the any clinical set up whilst recycling should be integral to overall waste management. However, reduction in the amount of waste can only be planned after thorough analysis of the clinical waste composition. On average, 30% of all dental waste has been reported to be from the packaging (Arora, Mittal, and Dogra 2017). Single‐use materials form a major proportion of clinical waste that could potentially be replaced with reusable items. Creating a system whereby accurate segregation and management of waste becomes integral part of health care provider's standard operative procedure is fundamental as it will increase the amount of recycled waste and reduce the amount of general waste produced.

James Cook University Dental (JCU Dental) clinic is an educational facility based in Cairns, Australia, where students provide comprehensive dental care to the northern Queensland community under supervision. JCU Dental has provided affordable dental treatment to more than 63,000 patients since 2012, proving to be a major contributor in the provision of oral health care for the population of Cairns and surrounding health districts. As a major oral health facility in the region, student clinicians, supervising clinicians and dental assistants facilitate provision of dental treatment to the general public, significant amounts of clinical waste are generated. To improve the environmental sustainability of the university clinic, an analysis of the waste and the current management procedures are needed to identify avenues for improvement. Furthermore, the findings could be utilized in conjunction with Kotter's change management theory, an eight‐step guide into implementing change (Duane et al. 2019).

Although previous studies on waste management in large medical health facilities exist, a limited number of studies have evaluated the contribution of the dental health sector in this context. Furthermore, current literature lacks research into larger dental clinics, with studies completed in one dental clinic unable to provide a generalized insight (Cannata et al. 1997; Farmer et al. 1997; Nabizadeh, Faraji, and Mohammadi 2014; Richardson et al. 2016). Furthermore, no studies have been conducted with a specific focus on a university educational facility, therefore possible avenues for improvement in the waste management procedures and protocols are yet to be explored. Hence, this study was conducted to analyze dental waste produced and identify avenues for reducing the environmental impacts of university dental clinics.

## 2 Materials and Methods

This project was designed as a quantitative study of dental clinical waste production in a large university clinic (James Cook University, Cairns Australia). This study protocol was approved (#H8154) by the James Cook University Human Ethics Committee (JCU HREC).

Fifty dental chairs, which accounted for equal number of patients and dental student clinicians along with 15 supervising clinicians were randomly selected to collate waste generated. All the above were considered participants since the data collection involved them during dental treatment at the university clinic conducted in 2021 (a single day in August) while analysis was completed by 2022. The inclusion of members of the Aboriginal and Torres Strait Islander community and children was dependent on the random number generator and patients involved on the day of data collection.

### 2.1 Recruitment and Enrolment

Recruitment occurred via a randomized number generator using Microsoft excel spreadsheet with numbers that correspond to each student's dental chair number. Potential participants (dental students and supervising clinicians) were informed of the proposed study commencing in the second half of the year, without specifying dates to avoid bias, through the University's learning management system. The details of the study were also announced in the clinic newsletter with regular reminders before clinical sessions through printed information sheets.

Dental assistants were designated to provide consent forms to dental students and supervising clinicians at the end of selected treatment sessions. Investigators of the research team were available for the dental student and supervising clinicians at the end of the session to verbally clarify any questions or concerns regarding the research and to collect signed consent forms. All dental students and supervising clinicians associated with randomly selected dental chair signed the consent form after the treatment was completed. Patients scheduled to be treated in these randomly selected chairs were provided information about the study, requested to sign consent forms before any dental treatment commenced and were advised to refrain from discussing about this project with student clinicians and supervising clinicians to avoid bias.

### 2.2 Data Collection

Due to recent COVID‐19 and associated regulatory requirement, a comprehensive risk assessment for the project was completed. All investigators donned full personal protective equipment (PPE) during the handling of waste. PPE was at the forefront of our precautions, with Level 2 Dental Masks, protective eyewear, gloves, and gowns always worn along with social distancing implemented at all stages of the research. After obtaining consent from dental students and supervising clinicians, clinical waste generated during dental treatment was collected and segregated by the five investigators depending on its category. All the categorized waste products were weighed using electronic scales and recorded against the dental procedures performed, for subsequent analysis.

### 2.3 Data Analysis

Data were analyzed using the Statistical Package for Social Sciences (SPSS version 25) software. Results were sorted into pie charts and bar graphs to compare the waste composition, as well as the total waste composition and usage of various materials. Further pairwise comparisons based on waste generated and treatment procedure performed was conducted. Data on waste generated was extrapolated to estimate annual student clinical waste generation and greenhouse gas emission.

## 3 Results

The contaminated waste from 50 patient treatment sessions from fourth‐year dental student clinicians were sorted into seven different categories: aprons, gloves, masks, sterile wrapping, plastics, cellulose, and miscellaneous, with the sharps considered separately. The plastics category included waste but was not limited to barriers, plastic cups, triplex tips, and bond or composite capsules. The cellulose waste group consisted of paper towels, gauze, cotton rolls, disinfectant wipes, bibs, and bib chains. The miscellaneous group included waste such as rubber dams, impression materials, and glass ionomer cement (GIC) capsules. GIC capsules were added to the miscellaneous group rather than the plastics category as most of the disposed capsules contained more than half of the unused material. Sharps bins were weighed, before any dental treatment, and subsequently weighed after dental treatment to deduce the weight of sharps used for a patient. The sharps category included burs, wedges, local anaesthetic carpules, needle tips, and matrix bands.

Types of dental procedures performed by dental students under supervision included examinations, periodontal (scaling and debridement), restorative (mostly composite restorations), fixed prosthodontics (crowns and bridges), removable prosthodontics (removable full and partial dentures, denture repairs), oral surgery (simple and surgical dental extraction), and endodontics (single root and uncomplicated multi‐rooted teeth). In some cases, more than one dental procedure was performed during the appointment, for example, examination followed by extraction. Student (clinician) to supervisor ratio was 6‐7:1 with up to two dental assistants available for 6 chairs, with their role limited to transfer materials for the students attending the patients.

Most disposed category of clinical waste was cellulose at a total of 5040 g across the 50 appointments with an average of 100.80 g per appointment (Table 1). As mentioned previously, the cellulose category consisted of items such as paper towels, gauze and cotton rolls which are absorbable and frequently in contact with saliva, water and other fluids which can potentially inflate the total mass to some extent. The second most disposed category was aprons with 4070 g total and 81.4 g average per appointment. Due to the changes brought in due to COVID‐19 clinicians needed to don new gowns every time they entered a bay since exposure to aerosol is anticipated and has heavily inflated the PPE usage and contributed to increased waste.

**Table 1:** Mass of disposed waste by category.

	Sharps	Aprons	Gloves	Masks	Sterile	Other plastics	Cellulose	Misc.
Mean (g)	29.3	81.4	56.20	17.20	24.00	48.80	100.80	25.80
Std. Deviation	41.858	50.589	30.848	12.583	17.350	38.921	65.725	42.599
Minimum (g)	0	0	5	5	5	5	5	0
Maximum (g)	8	205	170	55	60	175	325	200
Sum (g)	1465	4070	2810	860	1200	2400	5040	1290

The third most disposed waste category was gloves with 2810 g total and 56.20 g average, closely followed by the other plastics category, producing 2400 g total and 48.80 g. Sharps waste produced at a total of 1465 g (average 29.3 g). The sharps mass may be skewed as sorting through the sharps bin was not considered safe due to health and safety reasons. On average 25.8 g of miscellaneous waste was produced per appointment with a total of 1290 g. Sterile wrapping and masks had the two lowest average masses per appointment with 24 and 17.2 g, respectively.

It is worth noting that sterile wrapping is often disposed of before the patient treatment commences, or never enters the treatment area thus, has the greatest recyclability potential as it is not contaminated. Sterile wrapping consists of a cellulose‐based backing with a firm plastic film covering the instruments therefore the total mass was divided evenly between the plastic and cellulose categories. The aprons are made of a polymer material thus their mass was added to the plastic category. Finally, the mass of the masks disposed was added to the cellulose category. With these changes, the most disposed material was plastic, encompassing 37% of all waste with 7055 g total. Cellulose was the second most disposed material with a total weight of 6475 g and taking up 34% of total waste. Nitrile, sharps, and miscellaneous had the least total masses with 2810, 1465, and 1290 g respectively. Overall, the total mass of the 50 selected appointments for all categories of wasted was 19,354 g (Table 1).

**Figure 1:**
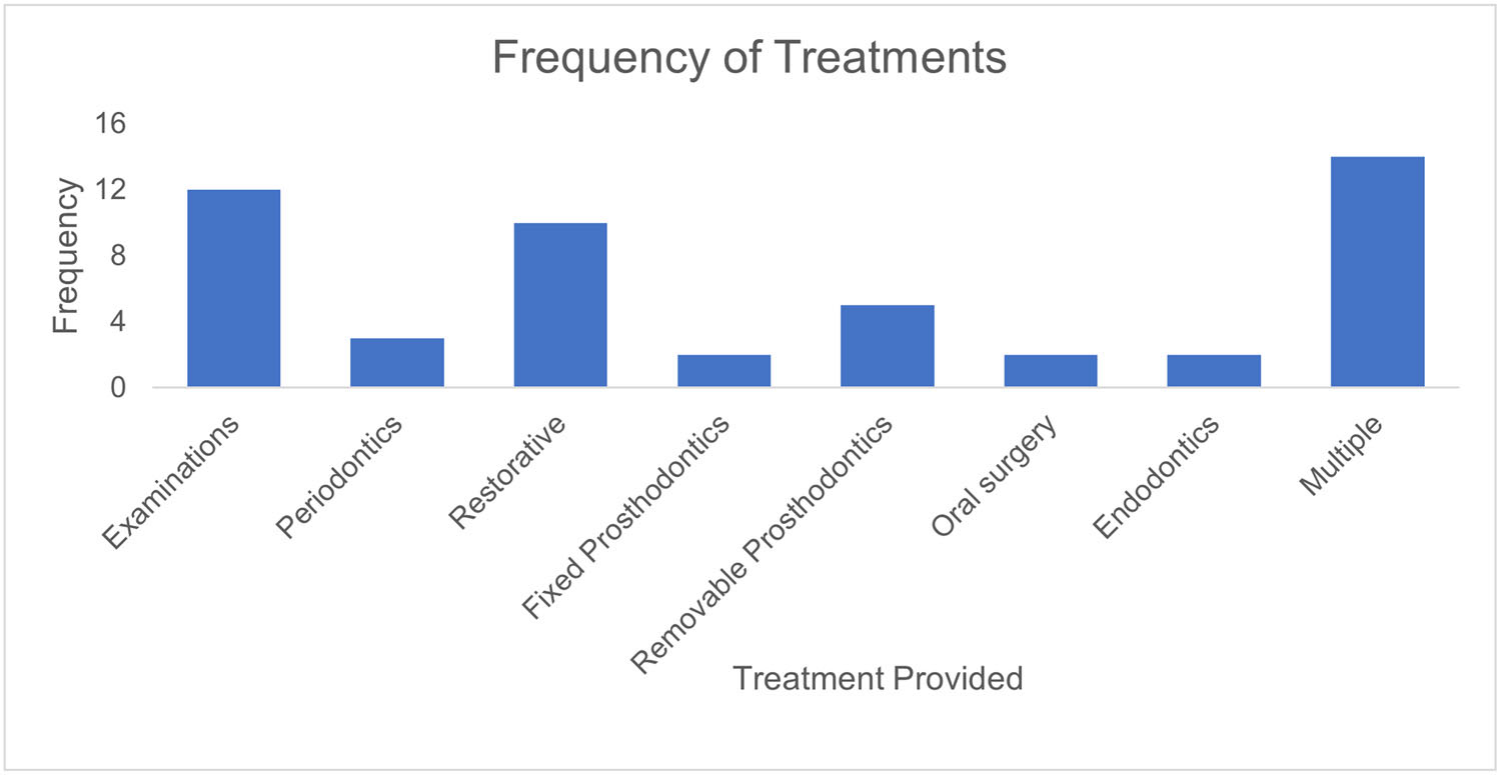
Frequency(s) of treatments performed.

The frequency of different treatments performed on the 50 selected patients is illustrated in Table 2 and Figure 1. The treatments performed consisted of examinations, periodontics, restorative, fixed prosthodontics, removable prosthodontics, oral surgery, endodontics, and a combination of multiple treatments. 14 appointments consisted of multiple treatments which was the highest frequency of treatment categories. The second most frequent treatment was examinations with 12 appointments followed by restorative procedures with 10 appointments. On the day of collection, five removable prosthodontic treatments were performed, and three patients received periodontal treatment. Fixed prosthodontics, oral surgery, and endodontics were performed in two occasions.

**Table 2:** Frequency of treatments performed.

Treatment performed	Occasions	Percent (%)
Examinations (e.g., comprehensive oral exams)	12	24
Periodontics (e.g., scaling and debridement)	3	6
Restorative (mainly composite and GIC fillings)	10	20
Fixed Prosthodontics (single crown or multi‐unit bridge)	2	4
Removable Prosthodontics (full and partial denture fabrication, denture repair)	5	10
Oral Surgery (simple extractions, surgical extractions)	2	4
Endodontics (Single rooted tooth or multi‐rooted tooth)	2	4
Multiple Treatments (e.g., examination and filling)	14	28

As shown in Table 3, there are vast disparities in the composition of waste produced across the different treatment categories. Fixed and removable prosthodontics had a considerably lower average of disposed sharps with 2.5 and 13.0 g, whereas oral surgery appointments had over four times the total average of sharps at 132.5 g. This is likely due to the multiple local anesthetic carpules used for extractions. Fixed prosthodontics had the highest mass of aprons and gloves disposed at 120.0 and 110.0 g, respectively. As fourth‐year dental students have limited experience in fixed prosthodontics, supervising clinicians would have provided close supervision, accounting for significantly higher use of aprons. There was no significant variation in the mass of masks disposed ranging from 11.0 g in removable prosthodontics to 25.5 g in restorative treatments. Restorative treatments also had the highest disposed amount of sterile wrapping at 42.5 g. There are many components in restorative treatments that add to sterile wrapping waste that are often not used in other treatments such as rubber dam clamps and frames, v‐ring matrix clamps and tools, shaping instruments, and retraction cord packers.

**Table 3:** Average mass (g) of waste by composition and treatment.

Treatment	Sharps (g)	Aprons (g)	Gloves (g)	Masks (g)	Sterile (g)	Other Plastics (g)	Cellulose (g)	Misc (g)
Examinations	36.25	66.25	47.50	12.5	12.5	28.75	100.42	9.58
Periodontics	33.33	71.67	38.33	11.617	30.00	51.67	33.33	0.00
Restorative	31.50	98.00	51.50	25.50	42.50	55.50	112.00	25.00
Fixed pros.	2.50	120.00	110.00	15.00	5.00	87.50	72.50	12.50
Rem pros.	13.00	67.00	57.00	11.00	16.00	44.00	102.00	27.00
Oral Surgery	132.50	20.00	27.50	12.50	32.50	75.00	70.00	7.50
Endodontics	30.00	75.00	75.00	22.50	32.50	52.50	95.00	55.00
Multiple	15.71	93.93	64.29	18.93	22.50	52.50	116.43	45.71

Examinations had the least plastic disposed at 28.75 g whereas fixed prosthodontics and oral surgery produced 87.5 and 75.0 g each. For each treatment, the mass of cellulose disposed was around 70–110 g except for periodontal treatments which produced the lowest average at 33.33 g. Endodontic treatments produced the highest mass of average miscellaneous items at 55 g followed by multiple treatments at 45.71 g. Endodontics likely produced the most miscellaneous waste due to the multiple rubber dams used to isolate and GIC capsules for temporary restorations. However, rubber dams are routinely used in restorative treatments such as composite fillings to achieve adequate moisture control. Notably, periodontal treatments had no miscellaneous waste.

Pairwise comparisons were conducted based on waste type and treatments to check for significant differences between groups using Friedman two‐way ANOVA with Bonferroni correction for multiple tests. As noted in Table 4, about 14 pairwise comparisons had significant difference in total waste generated during treatment before the Bonferroni correction for multiple tests was applied. However, the adjusted significance following post hoc remained statistically significant in six group comparisons with highest significance in examinations versus endodontics and multiple versus endodontics comparison. Additionally, pairwise comparison based on waste type showed similar trends, that is, 13 significant differences that reduced to four after post hoc correction was applied (Table 5). Notably, highest significance values were evident in masks versus aprons and masks‐cellulose comparisons after post hoc correction.

**Table 4:** Pairwise comparisons by treatment types.

Groups compared	Test statistic	Std. error	Std. test statistic	Sig.^a^	Adj. sig.^a^
Examinations‐Rem pros	−2.625	1.225	−2.143	0.032	0.898
Examinations‐Periodontics	−3.000	1.225	−2.449	0.014	0.401
Examinations‐Oral surg	−4.563	1.225	−3.725	< 0.001	0.005^b^
Examinations‐Fixed pros	−4.625	1.225	−3.776	< 0.001	0.004^b^
Examinations‐Endodontics	−5.688	1.225	−4.644	< 0.001	0.000^b^
Multiple‐Periodontics	2.625	1.225	2.143	0.032	0.898
Multiple‐Oral surg	4.188	1.225	3.419	< 0.001	0.018^b^
Multiple‐Fixed pros	4.250	1.225	3.470	< 0.001	0.015^b^
Multiple‐Endodontics	5.313	1.225	4.338	< 0.001	0.000^b^
Restorative‐Oral surg	−2.438	1.225	−1.990	0.047	1.000
Restorative‐Fixed pros	−2.500	1.225	−2.041	0.041	1.000
Restorative‐Endodontics	−3.563	1.225	−2.909	0.004	0.102
Rem pros‐Endodontics	−3.063	1.225	−2.501	0.012	0.347
Periodontics‐Endodontics	−2.688	1.225	−2.194	0.028	0.790

^a^ Only comparisons with significance (< 0.05) listed.

^b^ Significant difference after adjustment by the Bonferroni correction for multiple tests.

**Table 5:** Pairwise comparisons of groups by waste types.

Groups compared	Test statistic	Std. error	Std. test statistic	Sig^a^	Adj. sig.^a^
Masks‐Other plastics	−3.438	1.225	−2.807	0.005	0.140
Masks‐Gloves	3.750	1.225	3.062	0.002	0.062
Masks‐Aprons	4.625	1.225	3.776	< 0.001	0.004^b^
Masks‐Cellulose	−4.875	1.225	−3.980	< 0.001	0.002^b^
Misc‐Other plastics	3.000	1.225	2.449	0.014	0.401
Misc‐Gloves	3.313	1.225	2.705	0.007	0.191
Misc‐Aprons	4.188	1.225	3.419	< 0.001	0.018^b^
Misc‐Cellulose	4.438	1.225	3.623	< 0.001	0.008^b^
Sterile‐Gloves	2.625	1.225	2.143	0.032	0.898
Sterile‐Aprons	3.500	1.225	2.858	0.004	0.119
Sterile‐Cellulose	−3.750	1.225	−3.062	0.002	0.062
Sharps‐Aprons	−3.375	1.225	−2.756	0.006	0.164
Sharps‐Cellulose	−3.625	1.225	−2.960	0.003	0.086

^a^ Only comparisons with significance (< 0.05) listed.

^b^ Significant difference after adjustment by the Bonferroni correction for multiple tests.

To investigate the potential to reduce greenhouse gas emissions, the “What's in a bin” method by Richardson et al. was followed closely (Richardson et al. 2016). GHG conversion factors for waste disposal were gathered from the 2011 Guidelines to Department for Environment, Food, and Rural Affairs and Department of Energy and Climate Change's (Defra/DECC) Green House Gas Conversion Factors for Company Reporting (Richardson et al. 2016). Our results highlight those 120 g of recyclable sterile wrapping plastics were generated by one dental student treating one patient per day during a 5‐day working week in a typical university year considered at least 40 weeks for calculations (Table 6).

**Table 6:** Calculation of Green House Gas emission for sterile wrapping.

	GHG emissions	CO_2_e per university year
Disposing of sterile wrapping as clinical waste	0.00012 tonnes × 1833 kg CO_2_e per tonne = 0.21996 kgCO_2_e.	8.7984
Disposing of sterile wrapping as recycled waste	0.00012 tonnes × −302 kg CO_2_e per tonne = 0.03624 kg CO_2_e.	1.4496
GHG savings per year for 1 student treating 1 patient per day	10.248 CO_2_e	
GHG savings per year for 100 students treating one patient/day	1024.8 CO_2_e	

When the clinic runs at full capacity, with each chair used for treating one patient per day for 40 weeks, GHG saved a total of 1024.8 kg CO_2_e if sterile wrapping plastic is recycled. It is also important to note that these sterile wrapping plastics are not contaminated, as they are generally disposed of before treatment and hence can potentially be directly placed in recycling containers.

## 4 Discussion

The Australian Dental Association's statement outlines the need for minimized environmental impacts in dental care without reducing the safety or quality of treatment (ADA, updated 2023). Implementation of environmental sustainability has been classically described as 4 R's‐reduce, reuse, recycle, and rethink (Arora, Mittal, and Dogra 2017). This approach becomes more relevant in large facilities including university clinics, wherein rigorous infection control procedures are critical and consequently produce a large amount of waste.

Dentistry, as a field, focuses on promotion and maintenance of oral and overall health, however, simultaneously contributing substantially to environmental pollution through waste production, high energy utilization, and hence carbon emissions (Al‐Qarni et al. 2016). Analysis of clinical waste data allowed us to assess the potential for cost and environmental impact reductions, to assess contaminated waste composition and quantity, to analyze the sustainability and cost‐effectiveness of clinical waste management, and to investigate the current waste management procedures and increase its efficiency. Currently, all categories of waste, excluding clinical waste, are placed in a single general waste bin. Rebecca Allan, an expert in waste management, has highlighted the increased costs of failing to correctly segregate waste streams (Holland 2014). Incorrect practices, like paper towels in clinical waste, have been found to increase costs by threefold (Holland 2014). Along with correct segregation of waste, an introduction of a separate recycling bin at each dental unit would result in increasing potential for recyclable waste. The status quo inhibits the opportunity for large‐scale recycling to occur and increases the overall volume of general waste. Recycling on a large scale will reduce greenhouse gas emissions and overall pollutant generation, thereby improving sustainability. Similarly, programs such as Environdent offer recycling of dental instruments via scrap metal companies that can significantly reduce waste in the instrument category (Scherer 2018).

A study conducted involving a six‐chair dental practice exploring the nature and quantity of clinical waste concluded that their findings were inadequate to create incentives to suggest policy changes mainly due to the small sample size (Richardson et al. 2016). The current study involves waste data from 50 dental appointments. With a greater scope, a more accurate estimation of waste produced from all large dental clinics can be assessed. Following these studies, an action plan can be developed to allow for informative changes to be made to reduce waste, carbon emissions, and costs.

Alternative methods of waste management in large dental facilities need to be implemented to improve the sustainability of the clinic in the future. In our study, a total of 19 kg of waste was produced over only 50 appointments, it is essential to acknowledge potential measures aimed at reducing the overall waste production of a large‐scale dental clinic. Plastic cups are utilized during each procedure as a transport medium from dispensary to the dental chair. Other uses include hydration purposes and prophylactic hydrogen peroxide mouth rinse as per the COVID‐19 risk mitigation measure. Compostable cups are an alternative to plastic cups which decompose under 6 months after disposal and require 30% less total energy expenditure during production. Cellulose was the second biggest waste contributor over the study period at 34%. Metal bib chains instead of paper disposable chains are an alternative that would aim at reducing this category of waste. Furthermore, part of the sterile wrapping waste is cellulose‐based and therefore has the potential to be recycled.

Notably, several dental products used at dental practices have no green alternatives available. Despite the significant time and financial commitment required for incorporating environmentally friendly products, greater commitment by dentists and dental clinics will help increase the changes that are necessary (Holland 2014). Additional government funding and incentives could provide the much‐needed boost to move toward implementing changes to create long‐term environmental sustainability.

Multiple studies across the world have been conducted to explore the student and faculty members' awareness and perceptions on environmental sustainability in dentistry (Al‐Qarni et al. 2016; Gershberg et al. 2022; Jamal et al. 2023; Spaveras and Antoniadou 2023). Educating dental students about the sustainable approaches in dental practice is critical in achieving optimal and long‐term buy‐in for the measures in a university setting. A large cross‐sectional study involving 26 dental schools in Saudi Arabia environmental sustainability in dentistry reported that although none of the schools had the sustainability formally embedded in their curricula, more than 80% of the staff and students acknowledged the importance of sustainable approaches in dentistry (Jamal et al. 2023). Another similar study involving one dental school each from the United States and the United Kingdom also confirmed the lack of formal inclusion of sustainability in dental curricula (Joury et al. 2021). At least three recent studies have successfully incorporated interventions by embedding environmental sustainability including sustainable learning outcomes and assessments (Duane et al. 2021; Field et al. 2023; Leung et al. 2022). In addition to general dental practice, specialty‐level studies exploring opportunities for sustainability have also been reported in restorative dental materials, prosthodontics, orthodontics, and pediatric dentistry (Ahmed, Brierley, and Barber 2023; Duane et al. 2019; Lyne 2022; Shinkai et al. 2023; Smith et al. 2023). Additionally, specific life‐cycle studies on toothbrushes, interdental brushes, dental examination, dental amalgam, and root canal treatment have significantly enhanced the importance of sustainable approaches in practices to minimize environmental impact (Abed et al. 2023; Borglin et al. 2021; Byrne et al. 2022; Duane et al. 2020; Jamal et al. 2021; Lyne et al. 2020; Spaveras and Antoniadou 2023). Hence, creating clinical workforce with clear understanding of sustainability in dentistry is important in communicating the vision of an ecofriendly dental clinic (Duane et al. 2019). All dental practitioners, students, supervising clinicians, and dental assistants need to be informed on the sustainability vision and plan at the organization level. Additionally, protocols and information on accurate segregation of waste, the significance of carbon emissions created from dental waste, and individuals' role in implementing of change needs to be established.

There are some limitations identified in this study. The data collection was conducted from 50 chairs (out of 80 chairs) that were randomly selected without consideration for the treatment planned on the specific day of data collection. Additionally, in a university clinic, higher PPE use is expected, compared to clinical practice due to the need for clinical supervisors supporting the student clinicians. Particularly use of disposable impermeable gowns (on top of the scrubs normally used) were much higher in and immediately after the COVID‐19 period due the infection control protocols mandated by the Australian Dental Association and the Health districts. This may have partly skewed the quantity of wastes within the PPE category. Also, teaching clinics tend to perform a limited range of procedures under supervision and hence the observations noted in our study may not be directly scalable to general dental practices.

## 5 Conclusion

There are significant opportunities to improve clinical waste management in large dental practices to achieve environmental sustainability. As noted in our study, simple measures can include disposal of sterile wrapping as recyclable waste instead of clinical waste, potentially resulting in significant reduction of greenhouse gas emissions. Additional education of students and practitioners, government funding support, and incentives may provide much‐needed boost to implement changes on a larger scale to sustain efforts in achieving long‐term success in environmental sustainability practices within dentistry.

## Author Contributions

**Samuel Yeoh:** conceptualization, writing–original draft (lead), formal analysis, writing–review and editing (equal). **Yani Bourdamis:** conceptualization, writing–original draft (lead), formal analysis, writing–review and editing (equal). **Adam Saker:** conceptualization, writing–original draft, formal analysis (lead), writing–review and editing (equal). **Noah Marano:** methodology (lead), formal analysis, writing–review and editing (equal). **Liam Maundrell:** conceptualization, methodology, writing–original draft, writing–review and editing (equal). **Poornima Ramamurthy:** validation, resources, formal analysis, writing–review and editing (equal). **Dileep Sharma:** supervision, validation, resources, formal analysis, writing–review and editing (equal).

## Ethical Statement

The study was approved (Approval ID #H8154) by the Human Research Ethics Committee (HREC) of James Cook University.

## Conflicts of Interest

The authors declare no conflicts of interest.

## Data Availability

The data that support the findings of this study are available from the corresponding author upon reasonable request.
